# Posttransplant Lymphoproliferative Disorders

**DOI:** 10.1155/2012/230173

**Published:** 2012-04-17

**Authors:** Hazem A. H. Ibrahim, Kikkeri N. Naresh

**Affiliations:** ^1^Department of Histopathology, Hammersmith Hospital Campus, Imperial College Healthcare NHS Trust and Imperial College, London W12 0HS, UK; ^2^Department of Histopathology, Faculty of Medicine, Mansoura University, Mansoura, Egypt

## Abstract

Posttransplant lymphoproliferative disorders (PTLDs) are a group of diseases that range from benign polyclonal to malignant monoclonal lymphoid proliferations. They arise secondary to treatment with immunosuppressive drugs given to prevent transplant rejection. Three main pathologic subsets/stages of evolution are recognised: early, polymorphic, and monomorphic lesions. The pathogenesis of PTLDs seems to be multifactorial. Among possible infective aetiologies, the role of EBV has been studied in depth, and the virus is thought to play a central role in driving the proliferation of EBV-infected B cells that leads to subsequent development of the lymphoproliferative disorder. It is apparent, however, that EBV is not solely responsible for the “neoplastic” state. Accumulated genetic alterations of oncogenes and tumour suppressor genes (deletions, mutations, rearrangements, and amplifications) and epigenetic changes (aberrant hypermethylation) that involve tumour suppressor genes are integral to the pathogenesis. Antigenic stimulation also plays an evident role in the pathogenesis of PTLDs. Plasmacytoid dendritic cells (PDCs) that are critical to fight viral infections have been thought to play a pathogenetically relevant role in PTLDs. Furthermore, regulatory T cells (Treg cells), which are modulators of immune reactions once incited, seem to have an important role in PTLDs where antigenic stimulation is key for the pathogenesis.

## 1. Introduction

Post-transplant lymphoproliferative disorders (PTLDs) are a group of diseases that range from benign polyclonal to malignant monoclonal lymphoid proliferations. They develop as a consequence of immunosuppression. PTLDs are characterised by the following: they are usually derived from B cells with preferential presentation as non-Hodgkin's lymphoma (as against Hodgkin's lymphoma), usually originate in extranodal sites, rarely affect skin, behave aggressively, and frequently harbour the Epstein-Barr virus (EBV) genome. Whilst most are high-grade B-cell non-Hodgkin's lymphoma (NHLs), a few are classical Hodgkin's lymphomas. Rare cases have also been shown to be either of T-cell or NK-cell lineages [1, 2].

T-cell neoplasms constitute 10% to 15% of all PTLDs, and about 75% of T-cell PTLDs, have been shown to be negative for EBV and to behave more aggressively. T-PTLDs usually develop later than B-PTLDs and patients are less likely to respond to reduction in immunosuppression [3, 4].

The abnormal B cells in solid organ transplant recipients originate usually from those of the recipient, while in recipients of bone marrow transplant they are of donor origin [5, 6].

## 2. Onset, Frequency of Occurrence, and Risk Factors of PTLD

PTLDs are classified as either early onset lesions which develop within one year, or late onset lesions, which develop more than one year after transplantation [7, 8].

The occurrence of PTLD varies between different studies, but the overall frequency is less than 2% in transplant recipients [9]. It differs according to many factors such as the age of the patient, the organ transplanted, type and dosage regimen of immunosuppressive drugs, and the pretransplant EBV serostatus [10].

**(1) The age of the patient**

children are more prone to developing PTLDs as they are usually naïve for Epstein-Barr virus (EBV) infection [10].

**(2) The organ transplanted**

the frequency of PTLD differs according to the type of organ transplanted. (Table 1) summarizes the frequency of PTLDs in transplant recipients [6, 9].

**(3) Type and dosage regimen of immunosuppressive drugs**

It has been reported that the risk of developing PTLD increases with the use of certain drugs such as tacrolimus and OKT3, especially when they are combined [11]. Despite the fact that immunosuppressive drugs are an established risk factor, it is still not well-understood whether the risk is due to the cumulative dose or peak levels of immunosuppressive drugs. The cumulative dose, however, is more likely to be the incriminating factor [12].

**(4) The pretransplant EBV sero-status**

EBV-naive recipients, being incapable of initiating an EBV-specific cytotoxic T-lymphocyte (CTC) response, are more liable to develop PTLD [12, 13]. Nonexposure to EBV before transplantation remains the most important predisposing factor [13].

## 3. Clinical Presentation

The clinical manifestations vary from nonspecific symptoms in the form of fever, sweats, malaise, weight loss, and features of primary EBV infection in some patients, to sudden enlargement of tonsils, lymph nodes, or other extranodal lymphoid organs. Other organs such as the central nervous system, bone marrow, spleen, lung, small intestine, liver, and kidney may also be affected [10].

## 4. Pathological Features and Classification of PTLDs

Clinicopathologic features of major types of posttransplant lymphoproliferative disorders are summarised in Table 2. The classification of PTLDs is currently based on the WHO classification of lymphoid neoplasms (Table 3). Three main pathologic subsets/stages of evolution are recognised: early, polymorphic, and monomorphic lesions [3]. 

### 4.1. Early Lesions

Early lesions form one end of the spectrum of PTLD and mostly develop within one year after transplantation. These include two morphological types: plasma cell hyperplasia and infectious mononucleosis-like lesions. Early lesions more frequently involve tonsils, adenoids or lymph nodes than true extranodal sites. They do not invade or disturb the architecture of the affected tissue [3].

Plasmacytic hyperplasia shows numerous polytypic plasma cells and occasional immunoblasts. Infectious mononucleosis-like lesions show marked paracortical expansion by a cellular infiltrate composed of numerous immunoblasts and a mixed population of T cells and plasma cells. These lesions often show spontaneous regression or regress following reduction in immunosuppression [3]. Immunoblasts in infectious mononucleosis-like lesions frequently harbour EBV and express EBV-encoded RNA (EBER) or EBV-LMP-1. Early lesions rarely harbour clonal cytogenetic changes [14].

### 4.2. Polymorphic PTLDs

**Figure 1:**
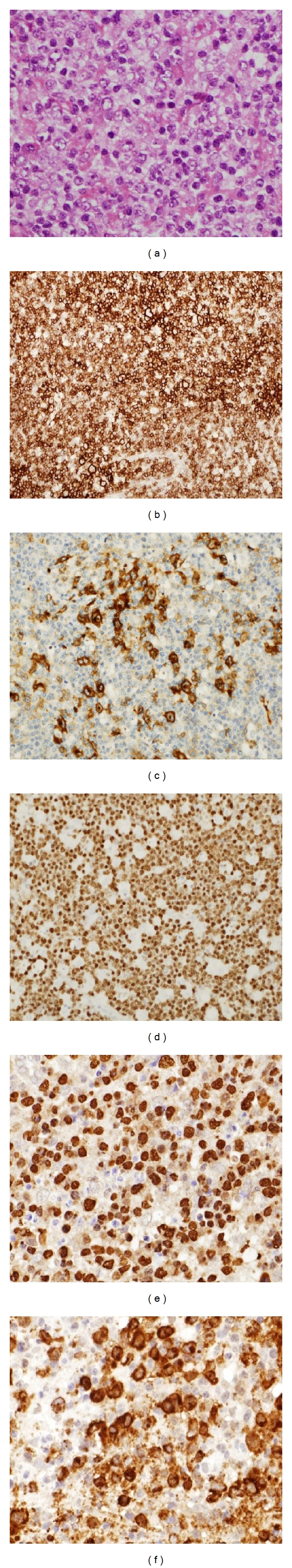
A typical case of polymorphic PTLD. (a) Infiltrate is a mix of plasma cells, small lymphoid cells and larger cells with nucleoli. The cells are positive for CD20 (b), CD30 (c), MUM1 (d), EBER (e), and EBV-LMP-1. Magnification: (b,d): ×100; (a,e,f): ×200.

Polymorphic PTLDs affect nodal and extranodal tissues and show loss of tissue architecture and necrosis. Polymorphic PTLDs are composed of a mixed population of immunoblasts, plasma cells, intermediate-sized lymphoid cells (incorporating a full range of B-cell morphology and differentiation), as well as occasional Hodgkin Reed Sternberg-like cells [15] (Figure 1). The B-cells are usually monotypic but may be polytypic. Nonetheless, a clonal pattern of *IgH* or episomal EBV genome is observed [16, 17]. The majority of the lesions exhibit EBV latency type II or III (expressing EBER and EBV-LMP-1 with variable expression of EBV-EBNA2 and other viral antigens). A variable proportion of cases show regression in response to reduction in immune suppression while other cases may progress and require chemotherapy [3].

### 4.3. Monomorphic PTLDs

Monomorphic PTLDs (mPTLDs) can be either of B cell or T-cell lineage and resemble the typical types of non-Hodgkin lymphomas (NHLs) seen in immunocompetent patients, and they are usually monoclonal. They disturb the tissue architecture and spread to other organs. They are classified according to the WHO classification of lymphomas in immunocompetent patients. Monomorphic B-PTLDs show features of different morphologic variants of diffuse large B-cell lymphoma (DLBCL) in immunocompetent patients (iDLBCL) (immunoblastic, centroblastic, or anaplastic), Burkitt's lymphoma (BL), or plasmablastic lymphoma (PL). Almost all cases display a clonal pattern of *IGH* rearrangement, and EBV-positive cases show episomal EBV genome. mPTLDs can be EBV-negative, tend to be more aggressive, and only rarely respond to a reduction in immune suppression [3, 18]. In addition, genetic alterations of 3q27, 8q24.1, and 14q32 have been described in monomorphic B-PTLDs [19]. The identification of similar cytogenetic alterations and clonal relationship between polymorphic PTLDs and mPTLD supports the hypothesis that PTLDs progress along a continuum from polyclonal early lesions to monoclonal mPTLD [3, 20]. 

Plasmablastic lymphomas (PBL), which were originally described in HIV-infected people affecting the oral cavity, may occur as a PTLD. Nearly 60–75% of cases of PBL are EBV associated [21–23].

There are only a few cases of PTLDs reported in the literature that demonstrate both B- and T-cell clones. In a recently published study, however, monoclonal expansion of T-cell population which seems to arise from CD8^+^ T cells has been found to occur frequently in B-PTLDs, and these clonal T-cell populations coexist with monoclonal B-cell population in B-PTLDs. However, these clonal T-cell expansions do not constitute a clinical T-cell lymphoma [24–26].

### 4.4. Hodgkin's Lymphoma-PTLD and HL-Like PTLD

The histological features of HL-type PTLD are similar to mixed cellularity or lymphocyte-depleted subtypes. The infiltrate is composed of scattered large pleomorphic mono- and binucleated Hodgkin/Reed-Sternberg giant cells in a background of small lymphocytes, B-immunoblasts intermingled with histiocytes, plasma cells, a few eosinophils, and neutrophils. The neoplastic cells are usually CD30^+^, and CD15^+^, EBER^+^, CD45^−^, OCT-2^−^/BOB1 [3]. In HL-like PTLD, the EBV^+^ cells are CD45^+^, CD20^+^, and CD15^+^ and EBV^+^ small and medium-sized lymphoid cells may be present. Distinguishing HL-PTLD from HL-like lesions is sometimes difficult, and it has been suggested that the latter are better diagnosed as either a polymorphic or monomorphic PTLD based on the overall morphological features [3]. 

## 5. Aetiology and Pathogenesis

**Figure 2:**
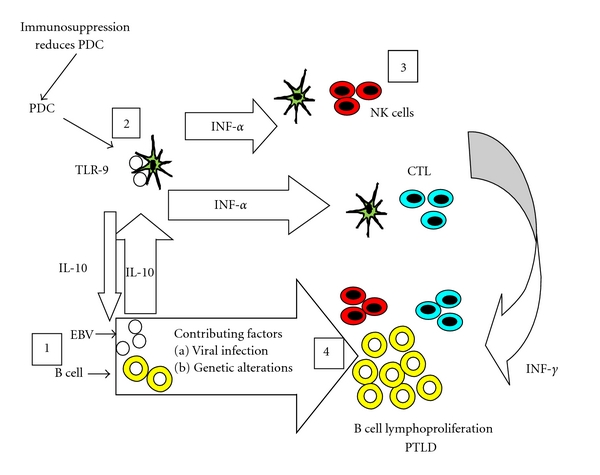
A proposed model of pathogenesis of EBV infection in the development of PTLDs in sold organ transplant recipients. CTL: cytotoxic T lymphocytes, IL-10: interleukin-10, INF-*α*: Interferon-*α*, NK cells: natural killer cells, PDC: plasmacytoid dendritic cells, TLR-9: toll-like receptor-9.

The pathogenesis of PTLD is multifactorial. EBV plays an important role in driving the proliferation of EBV-infected B cells. It is widely perceived, however, that it is not solely responsible for the “neoplastic” state, and that accumulation of different aberrations in protooncogenes and suppressor genes, and hypermethylation of suppressor genes are integral parts of the pathogenesis [27] (Figure 2).

### 5.1. Viruses

#### 5.1.1. EBV

EBV is an oncogenic double-stranded DNA virus that infects and persists in memory B cells. Two phases of EBV infection have been recognized. The lytic phase is characterized by the expression of all EBV proteins and active viral replication, leading eventually to cell death and the release of virions. The latency phase involves infection of lymphoid B cells via their CD21 receptors, resulting in the formation of EBV episomes and the expression of a limited number of viral proteins [28]. 

This results in persistence of the virus in the lymphoid cells and their progeny without destruction of the infected cell. LMP-1 and LMP-2 viral proteins are believed to act as oncogenes, allowing B cells to escape cell death and proliferate autonomously [28]. There are three different latency patterns that correspond to the differentiation stages of B cells. These patterns are thought to play a major role in protecting EBV-infected cells from immunosurveillance [29–31]. EBV-infected naive B cells expressing all latent antigens are said to have “type III latency.” Infected naive B cells enter the germinal centre where they multiply and form clones. They express EBNA1, LMP-1, and LMP-2, and this is known as “type II latency” [32]. However, some only express EBNA-1 and 2 as well as small noncoding Epstein-Barr RNAs (EBERS) and are said to have “type I latency” as seen in Burkitt's lymphoma [33, 34].

Most PTLDs are associated with EBV, but nonetheless a proportion (42% reported in one study) is EBV-negative, including 53% of the mPTLD cases [35, 36]. There is a debate as to whether EBV-negative PTLDs are in fact incidental lymphomas developing in transplant patients, or true PTLDs that can regress following reduction of the immunosuppression [37]. It has to be noted that the lack of identifiable EBV, based on EBER or LMP-1 staining, does not necessarily imply that EBV-DNA is absent in all of these cases, or that EBV did not play a role in the pathogenesis of the EBV-negative lymphoid proliferations [38]. It has been suggested that EBV-negative PTLD may develop as a result of “hit and run” oncogenesis as does EBV-negative classical Hodgkin lymphoma (cHL) [38, 39]. Chronic antigenic stimulation on the background of immune suppression is thought to play an essential role in the pathogenesis of EBV-negative PTLD [40].

B-PTLDs have been shown to be more frequently associated with type-A EBV genotype than type-B EBV [41, 42]. 

#### 5.1.2. HHV-8

HHV-8 is a gamma-herpes virus that, like EBV, infects B cells and acquires an episomal configuration in the nucleus and results in a state of latency. In the posttransplant setting, it has only been detected in cases of primary effusion lymphoma [43, 44]. In a recently published study, KSHV/HHV-8 was found to be consistently absent in PTLD [45].

### 5.2. Molecular Alteration of Cellular Genes

Different genetic alterations among PTLDs are summarised in Table 4.

#### 5.2.1. Microsatellite Instability

Lymphomas developing in immunocompetent patients are often characterized by relative genomic stability. In contrast, a small subset of PTLD is associated with microsatellite instability, which results from defects in DNA mismatch repair mechanisms [46]. These cases show mutations involving multiple genes, including *BAX* and *CASPASE5* (proapoptotic factors) and *RAD50* (a DNA repair gene) [27]. 

#### 5.2.2. Aberrant Somatic Hypermutation (ASHM)

B cells in the germinal centre (GC) are subjected to a physiological phenomenon known as “somatic hypermutation” (SHM), which involves the introduction of single nucleotide substitution into their *IgV* genes [47]. It involves not only the *IgH* gene but also nonimmunoglobulin genes such as *BCL6* and *Fas/CD95*. In more than 50% of DLBCLs, SHMs can also affect some proto-oncogenes such as *PAX5*, *PIM-1*, *RhoH/TTF*, and *c-MYC* gene, which are involved in the pathogenesis of lymphoid neoplasms including some cases of PTLD [47]. 

#### 5.2.3. Other Genetic Alterations

**BCL6 Gene**

The *BCL6* gene is located on chromosome 3q27 and encodes a transcriptional repressor [48]. *BCL6* rearrangement is very rarely seen in PTLDs, although it is the target of SHM in approximately 50% of PTLDs [14, 19].

**c-MYC Gene**

*c-MYC* gene is located on chromosome 8q24 and is the target of chromosomal breaks in most posttransplant Burkitt's lymphomas (PT-BL) [14, 49].

**BCL2 Gene**

*BCL2* gene, an antiapoptotic gene, is located on chromosome 18q21. Although the *BCL2* is amplified in a proportion of PTLDs, its rearrangement is a very rare event in PTLDs [14, 50].

**TP53 Gene**

The *TP53* gene is a tumour suppressor gene located on 17p13.1 and is mutated or deleted in a small proportion of mPTLDs (DLBCL) [16].

**IGH Gene**

*IGH* gene is located on 14q32 and breakpoints involving the gene are detected in a small proportion of PTLD and rarely in florid follicular hyperplasia in post-transplant setting [14].

**PAX5 Gene**

*PAX5* is the target of t(9;14)(p13;q32) as well as ASHM in a very small proportion of mPTLD (DLBCL) [51, 52]. A proportion of PTLDs has also been reported to have *PAX5* gene amplification [53].

**Other Chromosomal Changes**

Comparative genomic hybridisation (CGH) analysis of PTLDs highlights some genetic changes similar to those occurring in the lymphoma of immunocompetent patients; for example, gains of 3q27, 7q, 8q24, 12q, 12p, 18q21, and 21q and losses of 1p, 6q, 9p, and 17p13. In addition, PTLDs show losses of 4q, 17q and Xp that are not common in other lymphomas [50, 53]. It has been demonstrated that posttransplant-DLBCLs (PT-DLBCLs), with a frequency similar to iDLBCLs, show gains of chromosomes 5p and 11p. Moreover, deletions of 12p, 4p, 4q, 12q, 17p, and 18q are frequently seen in PT-DLBCLs [53]. The finding that iDLBCLs and a proportion of PTLDs (especially PT-DLBCLs) share some histogenetic and pathogenetic pathways is reinforced by the presence of recurrent chromosomal aberrations common to both PTLDs and iDLBCLs [54]. In addition, recurrent deletions on 11q25 and gains on 6q25.3 were observed in PT-BLs [53]. Rinaldi et al. [55] using high-density genome-wide SNP-based arrays, reported similar genomic complexity among PT-DLBCLs, HIV-DLBCLs, and iDLBCLs. Nonetheless, PT-DLBCLs displayed a genomic profile with distinctive features. It has been reported that the del(13q14.3) targets the locus coding for different noncoding RNAs [56]. The absence of del(13q14.3) in PT-DLBCLs is the most significant difference between PT-DLBCLs and iDLBCLs [55, 57]. Del(13q14.3) is thought to be involved in immunosurveillance escape in the view that immunodeficiency-related lymphomas including PTLDs lack del(13q14.3) [55].

PT-DLBCLs have *IgV* mutational status and gene expression profiles similar to post-GC B cells [3, 20]. Nonetheless, iDLBCLs of post-GC phenotype display genetic lesions that are different from PT-DLBCLs [55, 58, 59]. PT-DLBCLs have been reported to have gains of 1q, 11q, and of chromosome 7, in addition to losses at 17p (*TP53*) [55]. Compared with PT-DLBCLs, iDLBCLs were found to be more frequently associated with gains of 18q (BCL2 and NFATC1), and LOH at 6q21-q22 (approximately 7 Mb telomeric from PRDM1(BLIMP1)) and at 6p21.32-p21.33 (HLA-DR locus) [55].

Craig et al. [60] used Affymetrix HU133A GeneChips to show that EBV-positive mPTLDs overexpress several interferon-induced genes as compared to EBV-negative mPTLDs. Furthermore, EBV-negative PTLDs overexpress genes corresponding to the B-cell receptor signalling pathways and a group of proliferation-related genes. These suggest that EBV-negative PTLDs are biologically distinct from EBV-positive PTLDs and are more similar to iDLBCL [60].

When compared with EBV-negative PT-DLBCLs, EBV-positive PT-DLBCLs have been described as having less recurrent lesions. However, del(2p16.1) is common in both EBV-negative and positive PT-DLBCLs [60].

“Fragile sites” are regions with marked genomic instability, present throughout the genome, that are often the sites of DNA breakage in malignant tumours and in cells exposed to specific chemical agents [61]. PT-DLBCLs have been described to have frequent interstitial deletions at 1p32.2, 2p16.1, 3p14.2, 4p14, 14q13.2, 20p12.3, and 20q13.32. Some of these deletions involve fragile sites such as *FRA1B*, *FRA2E*, and *FRA3B*. Del(2p16.1) (*FRA2E*) is the most common aberration in PT-DLBCLs, and the involvement is significantly higher than in iDLBCLs [55]. Some viruses including EBV and HHV-8 have been shown to incorporate themselves into the host genome, mainly at fragile sites, resulting in local genomic instability at the insertion sites [55]. Iatrogenically immunosuppressed posttransplant patients are more susceptible to a wide range of viruses which could integrate into the genome, particularly at these fragile sites [55]. The dissimilar pattern of breakage at fragile sites reported in PT-DLBCLs and HIV-DLBCLs might be due to differences in the integration sites for various viruses [55].

#### 5.2.4. Epigenetic Alteration (DNA Hypermethylation)

Hypermethylation is an epigenetic phenomenon that alters the gene activity without changing its base sequences and is accomplished through DNA methyl transferase enzyme [62]. Aberrant hypermethylation (AH), which is a mechanism for tumour suppressor gene silencing alternative to deletion and/or mutation, has been implicated in the pathogenesis of lymphoproliferative disorders in the posttransplant setting [63].

**Hypermethylation of Death-Associated Protein Kinase (DAP-k)**

*DAP-k* is a serine-threonine kinase, which plays an important role in apoptosis triggered by TNF*α*, INF*γ*, and the FAS ligand. About 75% of mPTLDs display *DAP-k* hypermethylation [64].

**Hypermethylation of O6-Methylguanine-DNA Methyl-Transferase (MGMT)**

MGMT is one of the DNA repair genes that serves to protect against DNA damage. MGMT is methylated in nearly 75% and 93% polymorphic PTLDs, and mPTLDs respectively [65].

**Hypermethylation of P73**

*P73* is a tumour suppressor gene that bears some functional and structural resemblance to *TP53*. It plays a role in cell cycle regulation and apoptosis and is hypermethylated in about 20% mPTLDs [63].

**Hypermethylation of P16**

*P16 *is a tumour suppressor gene located on chromosome 9p21. It hinders the G1-S cell cycle transition by inhibiting the phosphorylation of Retinoblastoma protein. Martin et al. [66] described downregulation of P16/INK4a in subsets of mPTLDs (DLBCLs) that had an aggressive course but were not associated with EBV. There is a rare case report of an EBV-positive mPTLD (plasmablastic type) that showed *P16* hypermethylation [67].

**Hypermethylation of SHP1 Gene**

The *SHP1* gene is located on chromosome 12p13 and encodes the SHP1 protein. The protein is expressed in hematopoietic cells and potentiates its negative effect on cell cycle regulation by inhibiting the JAKs/STATs pathway. In B-lymphocytes, therefore, it inhibits proliferation, and its deficiency through AH results in overgrowth [68]. Cerri et al. [69] reported *SHP1* methylation in 76.5% of the PT-DLBCLs, 75% of the polymorphic PTLDs, 66% of the PT-BLs, and in a case of PT-myeloma.

### 5.3. Antigen Stimulation

Antigenic stimulation plays an important role in the pathogenesis of immunodeficiency-associated lymphomas. Under normal circumstances B cells express the B-cell receptor (BCR), and the loss of a functional receptor through the acquisition of mutations results in apoptosis [47]. It has been demonstrated that EBV, through expression of LMP2A which simulates a BCR, protects BCR-lacking GC B cells from death, leading to lymphoma development [47]. There are a few reports of the existence of EBV-negative PTLDs that lack expression of sIg, pointing to the possibility of as yet unidentified genetic mechanisms that may rescue EBV-negative, BCR-lacking lymphocytes [20]. Molecular signs of antigen stimulation are evident in a fraction of PTLDs that exhibit a functional BCR [47]. 

### 5.4. Role of Microenvironment

#### 5.4.1. Role of Plasmacytoid Dendritic Cells (PDCs)

PDCs are potent antigen-presenting cells that originate from the hematopoietic stem cells in the bone marrow under the effect of some cytokines, principally Flt3L [70]. In the posttransplant scenario, EBV-stimulated PDCs produce insufficient concentrations of IFN-*α*. Furthermore, the numbers of circulating blood PDC precursors are reduced in renal and cardiac transplant recipients. These are thought to play a significant role in the development of lymphoproliferative disorders [71, 72]. In addition, EBV-stimulated PDCs produce the immunosuppressive cytokine IL-10, thereby allowing the virus-infected B cells to escape immunorecognition [73]. IL-10 inhibits expression of costimulatory molecules, which in turn results in inability of monocytes and macrophages to activate T cells [74]. In addition, IL-10 suppresses the production of IFN-*α* and IFN-*γ* by PDCs, T cells, and NK cells. It also has an inhibitory effect on antigen-specific activation and proliferation [75]. PDCs numbers are increased in some malignant neoplasms including cutaneous T-cell lymphoma [76]. PDCs are markedly decreased in number and are qualitatively altered in non-Hodgkin lymphoma, compared with reactive lymph nodes [76]. However, in some cases of classical Hodgkin lymphoma (cHL), there are increased numbers of PDCs present which may be attributed to the cytokines released in the microenvironment of cHL [76]. The observation of PDC clusters in tumour samples suggests that PDCs may also play an important role in the pathogenesis of cutaneous marginal zone B-cell lymphoma [77]. Based on the finding of significantly higher numbers of PDCs in the tumour microenvironment of early lesions of B-PTLDs compared to polymorphic and monomorphic lesion, and in PT-DLBCL compared to iDLBCL, PDCs have been suggested to play a pathogenetically relevant role in PTLDs [78]. 

#### 5.4.2. Role of Treg Cells

Treg cells are CD4^+^ and CD25^+^ T lymphocytes that are a subset of immunoregulatory cells, and have the ability to suppress immune responses. There is a subpopulation of Treg cells which express CD8 and not CD4 [79]. When Treg cells undergo activation via their TCR, they inhibit the proliferation of CD4^+^ and CD8^+^ T lymphocytes, through the release of cytokines such as IL-10 and TGF-*β* [80, 81]. The intratumoural Treg cells have been shown to have an inhibitory effect on the production and release of perforin and granzyme B, which is necessary for the effector functions of CD8^+^ cells and cytotoxic T-cell-mediated lysis of tumour cells [82]. Treg cells are also known to have a direct effect on B lymphocytes and inhibit the production of immunoglobulins [83]. Treg cells can suppress the growth of some tumours in addition to their role in suppressing the antitumour immune response [84]. Higher numbers of Treg cells have been described as predictors of both improved survival in follicular lymphoma and therapeutic response [85]. Treg cells are found in higher numbers in tissue samples of B-cell lymphomas as compared to reactive lymph nodes or tonsils. This is thought to be due to the attraction of Treg cells to the tumour microenvironment through CCL22 secreted by the lymphoma cells [85, 86]. It has been previously shown that in recipients of solid organ transplants who are on multiple immunosuppressive drugs, the levels FOXP3^+^ Treg cells reduce in the peripheral blood, possibly due to redistribution into tissues and lymphoid organs [87]. The numbers of Treg cells in the tumour microenvironment of PTLDs have been shown to have no impact on patient survival [88]. 

## 6. Conclusion

PTLDs are group of diseases that range from benign polyclonal to malignant monoclonal lymphoid proliferations. Genetic and epigenetic alterations as well as viruses, notably EBV, contribute towards the development of PTLDs. Common genetic rearrangements which are frequent in immune competent lymphoma are rare in PTLDs. Microenvironment-resident PDCs and Treg cells are likely to play a critical role in the pathogenesis of PTLDs. Therefore, further studies investigating the cytokines secreted by PDCs and Teg cells are required to substantiate and further clarify their precise role in the pathogenesis of PTLD.

## Figures and Tables

**Table 1:** Frequency of PTLD in different types of transplants.

Organ transplanted	Reported risk of developing PTLD % and references
Kidney	1%
Liver	2–5%
Heart	2–5%
Lung	1.8–7.9%
Heart-lung	9.4%
Small bowel	up to 30%
Pancreas	2.1%
Bone marrow	<1%

**Table 2:** Clinicopathologic features of major types of posttransplant lymphoproliferative disorders.

	Early lesions (plasmacytic hyperplasia and infectious mononucleosis-like)	Polymorphic PTLD	Monomorphic PTLD
(i) Clinical features			
(a) Age	Children and young adults	All age groups	All age groups
(b) Organ involved	Tonsils or lymph nodes	Lymph nodes, GIT, lung or allograft	Lymph node, any extranodal site, including bone marrow
(c) Regression	Usually regress either with minimal reduction of immunosuppressive drugs or spontaneously	Some cases regress, others progress	Very rare. Most cases progress rapidly
(ii) Histopathological features			
(a) Tissue architecture	No or partial effacement	Nearly complete effacement	Complete effacement
(b) Nature of infiltrate	Comprised mainly of plasma cells and lymphoplasmacytoid cells in plasmacytic hyperplasia, and immunoblasts and plasmablasts in infectious mononucleosis-like lesion	Composed of a mixture of plasma cells, small lymphocytes, and large activated cells	Monotonous, similar to that of usual type B-cell NHL
(c) Atypia	Absent	Present/absent in large cells	Present
(d) Necrosis	Absent	Variable	Present (geographic)
(iii) Molecular features			
(a)* Ig* gene	Polyclonal in most cases	Usually monoclonal; may be oligo or polyclonal	Monoclonal
(b) EBV	Usually nonclonal	Clonal	Clonal
(c) Structural alterations of oncogenes and TSG	Usually absent	Usually absent	Usually present

Ig: immunoglobulin, EBV: Epstein-Barr virus, PTLD: posttransplant lymphoproliferative disorder, NHL: non-Hodgkin's lymphoma, TSG: tumour suppressor gene.

**Table 3:** Categories of posttransplant lymphoproliferative disorders.

(1) Early lesions
(a) Reactive plasmacytic hyperplasia
(b) Infectious mononucleosis-like lesions
(2) Polymorphic PTLD
(3) Monomorphic PTLD (classified according to lymphoma they resemble)
* B-cell neoplasms *
(a) Diffuse large B-cell lymphoma (DLBCL)
(b) Burkitt's lymphoma
(c) Plasma cell myeloma
(d) Plasmacytoma-like lesions
(e) Others*
* T-cell neoplasms *
(a) Peripheral T-cell lymphoma not otherwise specified
(b) Hepatosplenic T-cell lymphoma
(c) Others
(4) Classical Hodgkin's lymphoma-type (HL-PTLD) and HL-like PTLD**

*Indolent small B-cell lymphomas developing in transplant recipient are not included among the PTLD.

**HL-like PTLDs are better categorized either as a polymorphic or monomorphic PTLD based on the overall morphology.

**Table 4:** Summary of different genetic alterations among PTLDs.

Genetic alteration	Frequency
*BCL6 gene*	
(1) Rearrangement	Rare in PTLD
(2) SHM	50% of PTLD
*c-Myc gene rearrangement *	100% PT-BL
*BCL2 gene*	
(1) Rearrangement	Very rare in PTLD
(2) Amplification	A proportion of PTLD
*P53 gene mutation/deletion *	Small proportion of mPTLD
*Translocations involving IG genes *	A small proportion of PTLD. Rarely in florid follicular hyperplasia in posttransplant setting
*PAX5 gene*	
(1) Rearrangement	Very rare in PT-DLBCL
(2) SHM	Very rare in PT-DLBCL
(3) Amplification	A proportion of PTLD
*Chromosomal gains*	
(1) 3q27, 7q, 8q24, 12q, 12p, 18q21, 21q	
(2) 5p and 11p	PT-DLBCL = iDLBCL
(3) 6q25.3	Recurrent in PT-BL
(4) 1q, 11q, and of chromosome 7	PT-DLBCL
*Chromosomal loss*	
(1) 1p, 6q, 9p, and 17p13	Common to PTLD and lymphomas immune competent patients
(2) 4q, 17q, and Xp	In PTLD but not common in other lymphomas
(3) 12p, 4p, 4q, 12q, 17p, and 18q	Frequent in PT-DLBCL
(4) 11q25	Recurrent in PT-BL
(5) 2p16.1 (FRA2E)	30% of PT-DLBCL (both in EBV positive and negative cases)
(6) 17p	PT-DLBCL
*Aberrant hypermethylation of*	
(1) *MGMT *	75% pPTLD and 93% mPTLD.
(2) *DAP-kinase *	75% mPTLD
(3) *TP73 *	20% mPTLD
(4) *SHP1 *	~77% PT-DLBCLs, 75% pPTLDs, 66% PT-BLs
(5) *CDKN2A *	A small proportion of mPTLD

iDLBCL: immunocompetent diffuse large B cell lymphoma, mPTLD: monomorphic posttransplant lymphoproliferative disorders, pPTLD: polymorphic posttransplant lymphoproliferative disorders, PT-BL: posttransplant Burkitt lymphoma, PT-DLBCL: posttransplant diffuse large B cell lymphoma, SHM: Somatic hypermutation.
